# Glucagonoma and Glucagonoma Syndrome: One Center's Experience of Six Cases

**DOI:** 10.1089/pancan.2018.0003

**Published:** 2018-05-01

**Authors:** Jishu Wei, Xujun Song, Xinchun Liu, Zhenling Ji, Nithesh Ranasinha, Junli Wu, Yi Miao

**Affiliations:** ^1^ Pancreas Center, The First Affiliated Hospital of Nanjing Medical University, Nanjing, China.; ^2^ Department of General Surgery, Zhongda Hospital, Medical School of Southeast University, Nanjing, China.; ^3^ Jesus College, University of Oxford, Oxford, United Kingdom.

**Keywords:** glucagonoma, neuroendocrine tumors, diagnosis, therapy, prognosis

## Abstract

**Purpose:** Glucagonoma is an extremely rare neuroendocrine tumor arising from pancreatic islet cells. Although patients with glucagonoma manifest multiple typical symptoms, early diagnosis remains difficult due to the scarcity of this disease.

**Methods:** In this study, we retrospectively screened the database of the pancreas center of Nanjing Medical University. A total of six cases diagnosed as glucagonoma during the past 17 years were included. Their clinical characteristics and treatments were reviewed.

**Results:** The six patients consisted of four females and two males. Their median age at diagnosis was 48.7 years (range 35–77). The time from onset of symptoms to diagnosis of glucagonoma ranged from 1.3 months to >10 years. Common symptoms included necrotizing migratory erythema shown in six of six patients (100%), diabetes mellitus in five of six patients (83%), stomatitis in four of six patients (67%), and weight loss in four of six patients (67%). Plasma glucagon levels were elevated in all patients (range 245.6–1132.2 pg/mL; *n* < 200), and significantly declined after surgery (range 29–225.1 pg/mL; *n* < 200). Imaging studies revealed that three of six patients had metastasis at the time of diagnosis. All patients received surgical resection. The primary lesion, liver metastases, and involved organs were resected in all patients if present. The mean survival time was 5.7 years (range 3–10.4) from diagnosis and four of six patients died of this disease by the time of follow-up.

**Conclusion:** Our data suggest surgery is effective for symptom relief and can control the progress of glucagonoma. Early diagnosis and surgery are crucial for glucagonoma.

## Introduction

Glucagonoma is an extremely rare neuroendocrine tumor arising from pancreatic islet alpha cells with an incidence of ∼2.7/100 million in America.^1^ According to the World Health Organization classification, glucagonoma is a type of functional pancreatic neuroendocrine neoplasm (pNEN); as such, it should be diagnosed as a clinical syndrome. This pNEN causes a combination of symptoms named the glucagonoma syndrome.^2^ Classic symptoms include necrolytic migratory erythema (a typical skin disorder), diabetes, stomatitis, anemia, and other symptoms.^3^ It can also arise in patients with multiple endocrine neoplasia type 1, although less commonly.

Since the first reported case by Becker in 1942,^4^ many sporadic cases have been identified. In some instances, only a small number of patients had the symptoms of glucagonoma syndrome.^5,6^ This may have resulted from over diagnosis of immunoreactive glucagon fractions with reduced bioactivity.^7^ Here we present the cases of six typical patients from our center, in an effort to help clinicians better understand this rare disease.

## Methods

This study has been approved by the Ethics Committee of The First Affiliated Hospital of Nanjing Medical University. We screened our database for cases for the past 17 years. Patients with clinical presentations of glucagonoma syndrome, elevated plasma glucagon, and a pathological diagnosis of pancreatic islet cell tumor were included in this study. The medical records of the included patients were reviewed. Follow-up data, including patients' follow-up status and administration of other therapies, were acquired from hospital medical records or by phone interviews with the patients, relatives, or general practitioners from December 2016 to January 2017. In the case of death, the date of death was recorded.

## Results

### Clinical presentation

The six patients included four females and two males, and the median age at diagnosis was 48.7 years (35–77) (Table 1). The duration from initial symptom presentation to final diagnosis ranged from 1.3 months to >10 years (Table 2). Patients' symptoms are presented in Table 1. Necrotizing migratory erythema (NME) or diabetes was the first symptom in four of six patients. One patient had abdominal and back pain as his first symptom, originally misdiagnosed as pancreatic cancer with liver metastasis and hence received chemotherapy. Glucagonoma was diagnosed in this patient after diabetes and NME emerged. Another patient presented with abdominal pain as the first symptom and was erroneously treated for pancreatic cancer with gamma knife therapy. However, 5 years later this patient presented with NME from which a diagnosis of glucagonoma was confirmed. Other symptoms included stomatitis, weight loss, anemia, pitting edema of the legs, fatigue, poor appetite, impaired vision, hair loss, and diarrhea (Table 1). Among these symptoms, NME and impaired glucose tolerance or diabetes were present in all six patients and may be considered tumor-specific symptoms.

**Table 1.** **Patient Presentation, the First Department Visits, and Examinations**

Patient	1	2	3	4	5	6	Mean age/%
Male/female	F	M	F	F	M	F	
Age at diagnosis (years)	35	37	51	49	43	77	48.7
Year of diagnosis	2000	2001	2001	2010	2014	2015	
Symptoms at diagnosis							
NME	Y^a^	Y	Y^a^	Y^a^	Y^a^	Y	100%
Diabetes	N (but with IGT)	Y	Y^a^	Y	Y	Y	83%
Stomatitis^b^	Y	N	Y	Y	Y	N	67%
Weight loss	N	Y	NA	Y	Y	Y	67%
Anemia	Y	Y	Y	Y	Y	Y	100%
Other symptoms	Double lower leg pitting edema	Abdominal and lower back pain^a^	Fatigue and poor appetite	Both eyes decreased vision	Fatigue, hair loss, diarrhea, poor sleep	Abdominal pain^a^	
The first department visited	Dermatology	General surgery	Endocrinology department	Dermatology	Dermatology	NA	
Was skin biopsy diagnostic?	Y	ND	Y	NA	Y	ND	
Plasma glucagon (pg/mL)	245.6	359.8	873	1132.2	1570	500.9	
CgA (ng/mL)	ND	ND	ND	ND	360	ND	
NSE (ng/mL)	ND	ND	ND	ND	21.5	11.5	
Gastrin (pg/mL)	ND	ND	167.2	ND	ND	ND	
ESR	Normal	Normal	ND	ND	Normal	High	

Normal glucagon levels <200 pg/mL, normal gastrin levels <100 pg/mL.

^a^ First arose symptom.

^b^ Stomatitis here also includes glossitis and cheilitis.

NME, necrotizing migratory erythema; CgA, chromogranin A, normal <100 ng/mL; NSE, neuron-specific enolase, normal <16.3 ng/mL; ESR, erythrocyte sedimentation rate; IGT, impaired glucose tolerance; N, no; Y, yes; ND, not done; NA, not available.

**Table 2.** **Tumor Characteristics, Treatments, and Prognosis**

Patient	1	2	3	4	5	6
Time from symptoms to diagnosis (months)	4	26	1.3	31	240	72
Pancreatic tumor location	Neck and surrounding area	Body and tail	Body and tail	Body	Body and tail	Body and tail
Tumor size (cm)	4 × 3 × 3 and 4 × 3 × 2	7 × 5 × 5	3 × 1.5 × 1	5 × 3 × 2	5.5 × 4 × 4	10 × 8 × 6
Location of metastasis	NO	Liver, LN (3/3), gallbladder	liver, LN (1/2)	NO	NO (LN 0/4)	Liver, colon, diaphragm
TNM staging	IIb (T3 m, N0, M0)	IV (T3, N1, M1)	IV (T2, N1, M1)	IIb (T3, N0, M0)	IIb (T3, N0, M0)	IV (T4, Nx, M1)
Surgery	PD	1. DP + splenectomy 2. Cholecystectomy 3. Partial resection or electric ignition of liver metastases	1. DP + splenectomy 2. Hepatic metastasectomy	DP	DP	1. DP + splenectomy 2. Cholecystectomy 3. Part of the colon, liver, diaphragm resection of metastases
Glucagon immunostain	ND	ND	ND	+	+	+
Somatostatin analogues	Octreotide 100 μg × 3/day	Octreotide 100 μg × 3/day	ND	Octreotide 100 μg × 3/day	Octreotide 600 μg/day	Octreotide 100 μg × 3/day
Amino acid infusions	Y	Y	Y	Y	Y	Y
Chemotherapy	ND	GE, Capecitabine	ND	ND	ND	ND
Other treatments	ND	TACE, Hepatic metastases radiofrequency ablation	ND	ND	ND	Gamma knife
Glucagon after surgery (pg/mL)	69.8	NA	61.38	225.1	29	97.07
Symptoms improved	NA	2 Days after surgery and octreotide	1 Day after surgery	1 Day after octreotide	NA	NA
Survival (months)	44.5^a^	125^a^	36^a^	69^a^	30	26

Relevant TNM staging: T2 = tumor limited to the pancreas and size 2–4 cm; T3 = tumor limited to the pancreas and size >4 cm; M1 = distant metastasis. For any T, add (m) for multiple tumors.

Glucagon, normal range: <200 pg/mL.

^a^ Mean time from diagnosis to disease-related death.

NO, no metastasis was found by the surgery; LN, lymph nodes; PD, pancreaticoduodenectomy; DP, distal pancreatectomy; GE, gemcitabine; TACE, transarterial chemoembolization.

All six patients reported in our study had a delayed diagnosis of glucagonoma. Half of the cases (3/6) visited the dermatology department first, where psoriasis, eczema, or another skin disease was diagnosed and treated. One case visited the general surgery department first, where unfortunately pancreatic cancer was considered before referral to our center. Another case was admitted to the endocrinology department because diabetes mellitus was suspected. In each case, the department first visited was determined by the first symptom that arose in each patient.

### Examinations

Skin biopsies were obtained from four of six cases and pathologists diagnosed three of four of these with NME. All six patients had anemia upon investigation. Erythrocyte sedimentation rate was measured in four patients, but three of four patients' levels were normal. Serum plasma glucagon was significantly elevated (range 245.6–1132.2 pg/mL; normal <200 pg/mL) in all cases before surgery (Table 2). In our patient cohort, lesions were identified by computed tomography (CT) in three of six cases, by magnetic resonance imaging (MRI) in two of six cases, and by ultrasonography (US) in one of six cases.

### Therapy

Before being transferred to our center, one patient received gamma knife treatment in another institution and five of six patients were treated with injection of somatostatin analogues (SSA) in our hospital (Table 2). Skin symptoms improved in only one patient after using this drug, because only one of the patients received SSA before surgery and the other four received SSA after surgery. It is hard to tell whether the improvement of skin symptoms is the effect of SSA or surgery. All six cases received surgical resection in our center, involving pancreaticoduodenectomy (1/6) and distal pancreatectomy (DP) (5/6). Of the five patients who received DP, three also received combined hepatic metastasectomy (Table 2). Skin symptoms improved within 3 days after surgery in three cases. Patient 2 was referred to our center and received DP, splenectomy, cholecystectomy, and partial resection or cauterizing of liver metastases with electric knife. Pathology results confirmed malignant glucagonoma. Chemotherapy was applied after surgery. This patient received radiofrequency ablation for liver metastases 8 years after surgery.

### Pathological characteristics

The tumor was located in the pancreatic body and tail in five of six patients and in the pancreatic neck in one of six patients (Table 2). The primary lesion was single in five of six patients, whereas one of six patients had double primary lesions. Tumor size ranged from 3 × 1.5 × 1 cm to 10 × 8 × 6 cm. Metastases were found in three of the six patients during surgery. In patient 3, the metastasis occurred in the liver and lymph nodes. Patient 2 had gallbladder invasion, in addition to liver and lymph nodes metastases. Patient 6 had liver, colon, and diaphragm metastases. Liver metastases were often multiple. Pathologists diagnosed patients 2 and 3 with high-grade (poorly differentiated) pancreatic islet cell tumors, and diagnosed patient 1 with moderate differentiated pancreatic islet cell tumors. Patients 4, 5, and 6 were histopathologically diagnosed with pancreatic neuroendocrine tumors, and immunohistochemistry confirmed the final diagnosis of glucagonoma. The Ki-67 index was 3%–10% for patient 5, 1%–2% for patient 6, and was not mentioned in the other patients' pathological reports. In summary, two patients had low-grade malignant tumors, two had high-grade malignant tumors, patient 5 had a grade 2 (G2, moderate differentiated) tumor, and patient 6 had a grade 1 (G1, low-grade, well-differentiated) tumor.

### Follow-up

By the time of follow-up in January 2017, four of six patients had died of this disease. The mean time to disease-related death was 5.7 years (range 3–10.4) from diagnosis and 7 years (range 3–12.6) from the development of initial symptoms.

## Discussion

McGavran diagnosed the first case of glucagonoma by serum glucagon test in 1966.^8^ The male-to-female ratio of glucagonoma patients was reported as 0.8 with an average age of 52.5 years.^3^ However, our cohort included four females and two males, with an average age of 48.7 years. Glucagonoma diagnosis involves typical clinical symptoms, elevated serum glucagon level, and the presence of a pancreatic tumor. Typical symptoms include NME and diabetes; however, their low incidence in our cohort hindered diagnosis. Glucagon levels can vary from normal (usually <200 pg/mL) to as high as 14,300 pg/mL, and 66% to 81% patients have diabetes at the time of diagnosis.^2,9^ In accordance with this, in our cohort, glucagon levels varied from 245.6 pg/mL to 1570 pg/mL and 83% (5/6) patients had diabetes at the time of diagnosis.

All cases in our cohort were initially misdiagnosed, which is consistent with previous reports.^9^ NME was the first symptom in 67% (4/6) of patients but due to a lack of awareness of NME, half of our patients experienced a delayed NME diagnosis by the dermatologist. The duration between skin symptoms and diagnosis of glucagonoma can be as long as 11 years.^10^ For patients in our cohort, the average time from initial symptom presentation to diagnosis was 5.2 years. Diabetes is another key diagnostic symptom. Five of six of our patients had NME at diagnosis and the other had impaired glucose tolerance. In our cohort, stomatitis and weight loss were common (67%) and anemia appeared in 100% patients, but we believe these symptoms are unlikely to lead to a more rapid diagnosis of glucagonoma. Abdominal pain occurred in 33% (2/6) of our patients. Indeed, patient 2 was even treated for pancreatic cancer because he presented with both abdominal and lower back pain as his first symptom and a subsequent CT scan found a tumor in his pancreas.

Some studies report that glucagonoma patients may have venous thrombosis,^3^ vulvovaginitis,^11^ vomiting, fingernail deformity and fragility,^12^ depression,^13^ or nervousness.^14^ Neurological symptoms such as left-sided migraine headache^15^ and numbness or tingling^16^ were also found in reports. Reports of rare cases of accompanying heart disease also exist.^17^ The diagnosis of glucagonoma can be quite difficult.

In the past, CT scans were most effective at localizing glucagonomas.^3^ In our study, three cases were first confirmed by CT, two by MRI, and one by US. Glucagonoma can be definitively diagnosed by the presence of pancreatic tumor, typical symptoms, and elevated glucagon levels. Owing to socioeconomic reasons, none of our patients received somatostatin receptor scan (SRS). However, given the possibility of multiple tumors, we believe SRS is worth doing, despite the financial burden, because somatostatin receptor scintigraphy has been recommended as the best imaging technique for localization and staging for glucagonoma.^18^

Unlike previously reported cases,^9^ our study suggests that skin biopsy can lead to correct diagnosis. Two cases were quickly diagnosed as glucagonoma after skin biopsy. The classic histopathologic findings on skin biopsy are necrolysis of the upper epidermis with vacuolated keratinocytes.^16^ Characteristic histopathological features include psoriasiform epidermal hyperplasia, hypogranulosis, thickened stratum corneum, diffuse parakeratosis, and focal epidermal necrosis.^16^ NME was the characteristic symptom for patients in our cohort, as it was the first symptom in 67% (4/6) of our patients, and all six patients had NME at diagnosis. However, although NME can suggest glucagonoma, it is a nonspecific symptom since other disorders can also cause NME.^2,19^ This presents a real challenge for dermatologists.

Surgical resection is the only potentially curative treatment for glucagonoma and is the only way to control the syndrome.^20^ Over half of all glucagonomas are metastatic at the time of diagnosis. Synchronous resection of liver metastasis provides a more favorable outcome.^21^ Liver transplantation is controversial and sometimes may be effective in treating unresectable liver metastasis.^22^ Whether patients would benefit from tumor debulking or chemotherapy remains to be elucidated.^9,22^ Surgical resection is safe in a high-volume pancreas center and is associated with good long-term survival. To the best of our knowledge, perioperative mortality in present glucagonoma reports is low. Metastasis is a significant predictive factor for survival of patients with neuroendocrine tumors of pancreatic origin.^23^ The longest survival time reported in the literature is 24 years with an unresectable recurrence.^24^

Soga and Yakuwa report that glucagonoma usually locates in the pancreatic tail and 51.4% patients had metastasis, predominantly in the liver and lymph nodes. Extrapancreatic primary glucagonoma is so rare that only 3 cases have been reported out of 407 glucagonoma patients.^3^ More than 96% of patients had positive glucagon immunohistochemistry staining. The results of our study are consistent with those of Soga and Yakuwa In our cohort, the tumor was located in the pancreatic body and tail in five of six patients. Three patients (2, 3, and 6) had metastasis and three patients (4, 5, and 6) had positive glucagon immunohistochemistry staining.

In 2011, Eldor et al.^9^ reported six patients diagnosed with glucagonoma for a 25-year period. The median survival time for these patients was 6.25 years from diagnosis and 8 years from initial symptoms.

## Conclusion

Accurate diagnosis is the greatest challenge facing glucagonoma treatment. Currently, surgery is the best and most effective treatment option available for glucagonoma.

## Abbreviations Used

CT: computed tomography
DP: distal pancreatectomy
MRI: magnetic resonance imaging
NME: necrotizing migratory erythema
pNEN: pancreatic neuroendocrine neoplasm
SRS: somatostatin receptor scan
SSA: somatostatin analogues
US: ultrasonography
